# Vascular Response after Directional Coronary Atherectomy for Left Main Bifurcation Lesion

**DOI:** 10.1155/2021/5541843

**Published:** 2021-12-14

**Authors:** Norihiro Kobayashi, Masahiro Yamawaki, Mana Hiraishi, Shinsuke Mori, Masakazu Tsutsumi, Yohsuke Honda, Toshiki Chishiki, Kenji Makino, Shigemitsu Shirai, Masafumi Mizusawa, Kohei Yamaguchi, Takahide Nakano, Kaori Abe, Tomoya Fukagawa, Toshihiko Kishida, Yoshiaki Ito

**Affiliations:** Department of Cardiology, Saiseikai Yokohama City Eastern Hospital, 3-6-1 Shimosueyoshi, Tsurumi-ku, Yokohama, Kanagawa 230-8765, Japan

## Abstract

**Aims:**

To evaluate the vascular response after directional coronary atherectomy (DCA) for left main (LM) bifurcation lesion.

**Methods:**

This study was a retrospective, single-center study enrolling 31 patients who underwent stentless therapy using DCA followed by drug-coated balloon (DCB) angioplasty for LM bifurcation lesion. We compared intravascular ultrasound (IVUS) findings before and after DCA.

**Results:**

After DCA, the lumen and vessel areas significantly increased, whereas the plaque area (PA) and %PA were significantly reduced. When the lesions were divided into small vessel and large vessel groups using the median value of the vessel area, the maximum balloon pressure of the DCA catheter was greater in the large vessel group. Changes in the lumen and vessel areas were also significantly greater in the large vessel group. On the other hand, the changes in PA and %PA were similar between groups.

**Conclusions:**

The main vascular responses associated with lumen enlargement after DCA were plaque reduction and vessel expansion. Contribution of vessel expansion to lumen enlargement was larger than the effect of plaque reduction in large vessel lesions.

## 1. Introduction

Percutaneous coronary intervention (PCI) is an established option for the treatment of left main (LM) coronary artery disease [1]. However, complex stenting is sometimes inevitable in cases of LM bifurcation lesions and its efficacy has not been proven compared with that of the simple stent strategy [2]. Previous studies have reported the efficacy of directional coronary atherectomy (DCA) to avoid complex stenting in bifurcated lesions and to reduce restenosis of the left circumflex artery (LCX) ostium after single stent implantation for LM bifurcation lesions [3, 4]. Once the DCA catheter became commercially unavailable, a novel, improved DCA catheter (ATHEROCUT, Nipro Corporation, Osaka, Japan) was developed and became commercially available in Japan in 2015. Recently, the efficacy of the stentless strategy for LM bifurcation lesions using drug-coated balloon (DCB) angioplasty after DCA was reported [5]. Adequate lumen enlargement is important to achieve maximum efficacy of the stentless strategy using DCA followed by DCB angioplasty. The percentage plaque area (%PA) is a popular indicator used to determine the optimal endpoint of DCA. The main mechanism of lumen enlargement in DCA is plaque reduction [6–8], and increasing the balloon pressure of the DCA catheter further enhances plaque reduction [9]. On the other hand, balloon angioplasty is known to induce vessel expansion which is also one of the mechanisms of lumen enlargement [10, 11]. During DCA in LM bifurcation lesions, high balloon pressure of the DCA catheter is frequently required for large vessel lesions because the large plaque burden must be debulked. Therefore, the efficacy of vessel expansion, as well as the efficacy of plaque reduction, appears to increase for large vessel lesions. However, thus far, little is known regarding the vessel response after DCA in LM bifurcation lesions. We assessed intravascular ultrasound (IVUS) findings after DCA for LM bifurcation lesions in order to evaluate the vascular response.

## 2. Materials and Methods

### 2.1. Patient Population

Between April 2016 and October 2019, 58 patients who underwent PCI with DCA for LM bifurcation lesions were retrospectively identified. Of these, 27 patients were excluded; 18 underwent implantation of drug-eluting stents (DESs) after DCA, two underwent DCA alone, and seven underwent DCA for proximal stent edge restenosis at the left anterior descending artery (LAD) ostium. Finally, 31 patients who underwent stentless therapy with DCA followed by DCB angioplasty for de novo LM bifurcation lesions were enrolled. Among these, one patient underwent DCA followed by DCB angioplasty for both the LAD ostium and the left circumflex artery (LCX) ostium; therefore, we analyzed IVUS findings after DCA for 32 lesions in 31 patients.

Indications for DCA for LM bifurcation lesions were as follows: (1) stable angina pectoris with LM bifurcation lesion involving the distal LM trunk, the LAD ostium, or the LCX ostium; (2) a reference diameter of >2.5 mm in the main branch using visual estimation; and (3) IVUS findings suitable for DCA (no lipid-rich plaque, no thrombus, no severe superficial calcification, and plaque location to be debulked by DCA was accurately evaluated using IVUS). The exclusion criteria were as follows: (1) unstable angina pectoris and myocardial infarction, (2) poor patient's general condition and renal insufficiency (Cr > 1.5 mg/dL), (3) severe angle lesion, and (4) angiographic severe calcified lesion. This study was approved by the institutional review board of our hospital and complied with the Declaration of Helsinki. Written informed consent was obtained from all patients for both the procedure and subsequent data collection.

### 2.2. Procedure and Follow-Up

All PCIs were performed via the femoral artery using an 8Fr sheath introducer and 8Fr guiding catheter. During the procedure, the activated coagulation time was maintained at >300 s with administration of heparin. We carefully evaluated plaque distribution and plaque characteristics using IVUS after crossing the lesion with a conventional guidewire. We decided to perform DCA after plaque distribution to be debulked was adequately evaluated using IVUS and when there were no lipid-rich plaque, thrombus, and severe superficial calcification. The ATHEROCUT (Nipro Corporation, Osaka, Japan) was used for all lesions, and size selection was dependent on the reference diameter of IVUS. DCA was initiated with low balloon pressure (0 or 1 atm) and gradually increased based on the IVUS findings. We repeated IVUS evaluation after several cuts of DCA and again repeated to obtain residual %PA < 60% when possible [3]. The performance of the stentless strategy was decided after careful evaluation of the IVUS and angiographic findings by experienced operators. DCB angioplasty using SeQuent Please (Nipro Corporation, Osaka, Japan) was performed after DCA when IVUS revealed that there were no large residual plaque burden, no huge dissection, and no hematoma formation. The size of DCB was selected according to the reference lumen diameter by IVUS, and the balloon inflation time was 30 s with nominal pressure. Dual antiplatelet therapy with 100 mg/day aspirin and either 75 mg/day clopidogrel or 3.75 mg/day prasugrel was administered before the procedure and continued for 3 months following the procedure. Complications during the procedure and procedure-related major events during hospitalization including death, emergent target lesion revascularization (TLR) and coronary artery graft bypass, myocardial infarction, and access site complications were recorded. Myocardial infarction was defined as any postprocedural creatine kinase elevation of >2 times the normal level. All patients were followed up at 30 days after discharge and every 2 to 3 months subsequently. Follow-up coronary angiography was scheduled at 9 to 12 months after the procedure. TLR at 12 months and a major adverse cardiac event (MACE), defined as a composite of cardiac death, myocardial infarction, and any repeat revascularization at 12 months were investigated.

### 2.3. Quantitative Coronary Angiography Analysis

Quantitative coronary angiography (QCA) analysis was performed using the computer-based software (Heart II ver 2.0.2.3, GADELIUS) before the procedure, after the procedure, and at follow-up examinations using a guiding catheter to calibrate the magnification. Optimal views of the lesions were obtained at baseline, and the same projection angle was used at follow-up. Independent physicians who were blinded to all clinical information analyzed the minimal lumen diameter (MLD), reference diameter (RD), lesion length, and percent diameter stenosis (%DS). The acute gain was defined as the increase in MLD after PCI; late lumen loss was defined as the difference between the postprocedural MLD and MLD at follow-up.

### 2.4. Intravascular Ultrasound Analysis

All IVUS procedures were performed using commercially available IVUS catheters (OptiCross™; Boston Scientific, or ViewIT; Terumo) with automatic pull-back at a rate of 0.5 mm/s. At the lumen site where the lumen area was the smallest, the lumen diameter, lumen area, vessel area, and %PA were analyzed. PA was defined as the vessel area minus the lumen area. %PA was defined as (vessel area minus lumen area) × 100/vessel area. The changes in the vessel area, the lumen area, PA, and %PA after DCA were defined as postprocedure minus preprocedure values for the vessel area (Δ vessel area), lumen area (Δ lumen area), PA (Δ PA), and %PA (Δ %PA), respectively. These measurements were compared between small vessel and large vessel lesions, which were determined based on the median value of the vessel area. The incidence of hematoma, intimal dissection, and medial dissection was recorded. All images were analyzed using computerized planimetry software (echoPlaque; INDEC Medical Systems, Los Altos, CA, USA) by independent physicians who were blinded to all clinical data.

### 2.5. Statistical Analysis

Data are shown as numbers with percentages or means ± standard deviations. Comparisons of categorical variables were performed using Fisher's exact test. Comparisons of continuous variables were performed using Student's *t*-test or the Mann–Whitney *U* test. The Spearman rank correlation method was applied to estimate correlations between continuous variables. All *P* values were two-sided, and *P* values of <0.05 were considered statistically significant. All analyses were performed using SPSS software (version 19; IBM-SPSS, Chicago, IL).

## 3. Results

### 3.1. Patient and Lesion Characteristics

Patient and lesion characteristics are summarized in Table 1. A total of 31 patients with LM bifurcation lesions (mean age: 70 ± 10 years; male: 94%; diabetes mellitus: 26%; hemodialysis: 3%) were enrolled. DCA was performed more frequently for the LAD ostium (68%), followed by both distal left main trunk and the LAD ostium (17%). One patient (3%) with a true bifurcation lesion (Medina 1, 1, 1) underwent DCA for the distal LM trunk, LAD ostium, and LCX ostium.

### 3.2. Procedure Results

The procedure results are presented in Table 2. ATHEROCUT type *L* was the most frequently used (91%). The total number of cuts was 28 ± 17, and the maximum balloon pressure of the DCA catheter was 3.5 ± 1.3 atm. When lesions were divided into small vessel and large vessel groups according to the median value of the vessel area (14.9 mm^2^), the maximum balloon pressure was significantly higher in the large vessel group than in the small vessel group (2.8 ± 0.9 atm vs. 4.1 ± 1.3 atm, *P*=0.014). All lesions underwent DCB angioplasty after DCA with diameter 3.3 ± 0.4 mm and balloon pressure 8.3 ± 2.9 atm. In the QCA analysis, MLD and %DS were significantly improved after the procedure (MLD: 1.3 ± 0.4 mm vs. 3.4 ± 0.9 mm, *P* < 0.001; %DS: 63.3% ± 10.6% vs. 12.2% ± 7.9%, *P* < 0.001) (Table 3). There were no complications during the procedure and no procedure-related major events during hospitalization (Table 2).

### 3.3. IVUS Findings during DCA

**Figure 1:**
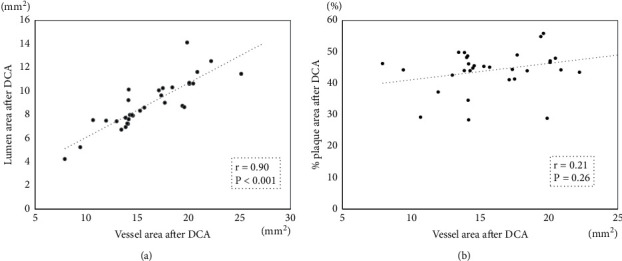
(a) Correlation between the lumen area after DCA and the vessel area after DCA. (b) Correlation between %PA after DCA and the vessel area after DCA.

**Figure 2:**
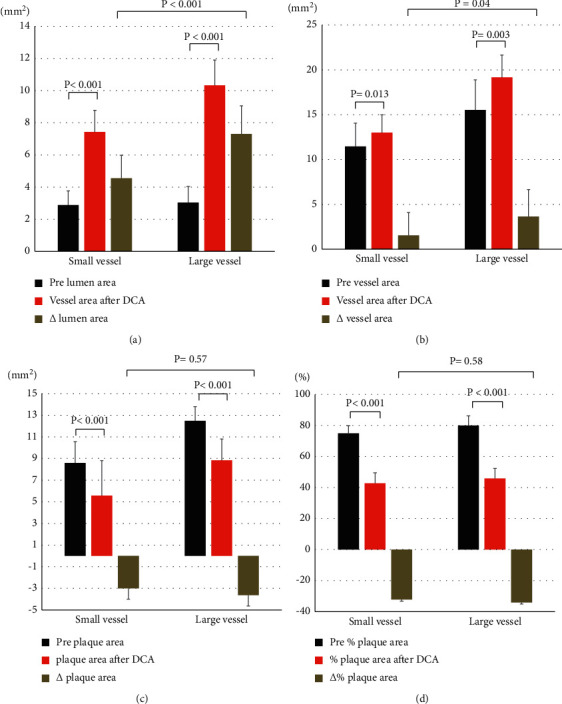
(a) Comparison of the lumen area before DCA, lumen area after DCA, and change in the lumen area between the small vessel and large vessel groups. (b) Comparison of the vessel area before DCA, vessel area after DCA, and change in the vessel area between the small vessel and large vessel groups. (c) Comparison of PA before DCA, PA after DCA, and change in PA between the small vessel and large vessel groups. (d) Comparison of %PA before DCA, %PA after DCA, and change in %PA between the small vessel and large vessel groups.

**Figure 3:**
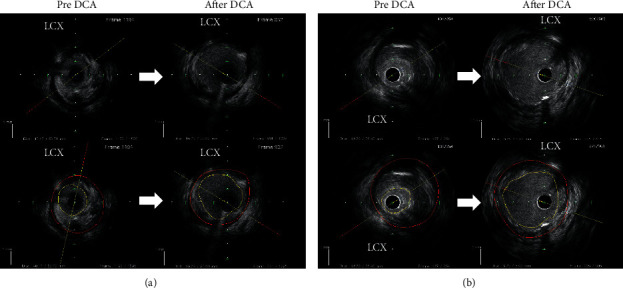
(a) IVUS findings before and after DCA in small vessel lesions. The red line represents the vessel area, and the yellow line represents the lumen area. The lumen area, vessel area, PA, and %PA before DCA were 2.7 mm^2^, 12.6 mm^2^, 9.9 mm^2^, and 78.5%, respectively. The lumen area, vessel area, PA, and %PA after DCA were 8.3 mm^2^, 14.9 mm^2^, 6.6 mm^2^, and 44.5%, respectively. The Δ lumen area, Δ vessel area, Δ PA, and Δ %PA were 5.6 mm^2^, 2.3 mm^2^, −3.3 mm^2^, and −34.0%, respectively. (b) IVUS findings before and after DCA in large vessel lesions. The red line represents the vessel area, and the yellow line represents the lumen area. The lumen area, vessel area, PA, and %PA before DCA were 2.3 mm^2^, 14.8 mm^2^, 12.6 mm^2^, and 84.8%, respectively. The lumen area, vessel area, PA, and %PA after DCA were 10.6 mm^2^, 19.8 mm^2^, 9.2 mm^2^, and 46.5%, respectively. The Δ lumen area, Δ vessel area, Δ PA, and Δ %PA were 8.3 mm^2^, 5.0 mm^2^, −3.4 mm^2^, and −38.3%, respectively.

IVUS findings during DCA are summarized in Table 4. Both lumen and vessel areas became significantly larger after DCA (lumen area: 3.0 ± 0.9 mm^2^ vs. 8.9 ± 2.1 mm^2^, *P* < 0.001; vessel area: 13.5 ± 3.6 mm^2^ vs. 16.1 ± 3.8 mm^2^, *P*=0.004). Both plaque area and %PA significantly decreased after DCA (plaque area: 10.5 ± 3.3 mm^2^ vs. 7.2 ± 2.3 mm^2^, *P* < 0.001; %PA: 77.5% ± 6.1% vs. 44.3% ± 6.7%, *P* < 0.001). There was a positive correlation between the lumen area after DCA and the vessel area after DCA (*r* = 0.90, *P* < 0.001). However, there was no correlation between %PA after DCA and the vessel area after DCA (*r* = 0.21, *P*=0.26) (Figures 1(a) and 1(b)). Δ lumen area and Δ vessel area were significantly larger in the large vessel group than in the small vessel group (Δ lumen area: 4.6 ± 1.4 mm^2^ vs. 7.3 ± 1.8 mm^2^, *P* < 0.001; Δ vessel area: 1.6 ± 2.5 mm^2^ vs. 3.7 ± 3.0 mm^2^, *P*=0.04) (Figures 2(a) and 2(b)). On the other hand, Δ PA and Δ %PA were similar between the small vessel and large vessel groups (Δ PA: −3.0 ± 1.6 mm^2^ vs. −3.6 ± 3.8 mm^2^, *P*=0.54; Δ %PA: −32.2% ± 7.9% vs. −34.1% ± 10.9%, *P*=0.58) (Figures 2(c) and 2(d)). Intimal dissection was observed in five lesions (15.6%); however, there was no medial dissection and hematoma formation.  Figure 3 shows representative IVUS findings before and after DCA in small and large vessel lesions. For small vessel lesions, Δ lumen area, Δ vessel area, Δ PA, and Δ %PA were 5.6 mm^2^, 2.3 mm^2^, −3.3 mm^2^, and −34.0%, respectively (Figure 3(a)). For large vessel lesions, Δ lumen area, Δ vessel area, Δ PA, and Δ %PA were 8.3 mm^2^, 5.0 mm^2^, −3.4 mm^2^, and −38.3%, respectively (Figure 3(b)).

### 3.4. Follow-Up Results

Angiographic follow-up was performed for 28 patients (angiographic follow-up rate: 90.3%). At follow-up coronary angiography, MLD and %DS were similar to those after the procedure (MLD: 3.3 ± 1.1 mm vs. 3.4 ± 0.9 mm, *P*=0.78; %DS: 15.4% ± 15.3% vs. 12.2% ± 7.9%, *P*=0.32) (Table 3). TLR at 12 months occurred in one patient (3.2%), and no MACE other than TLR was observed at 12 months. The only TLR case was LM bifurcation lesion with Medina classification (0, 1, 0). DCA was performed for the LAD ostium; however, post-%PA was 55.9% which was the largest in the enrolled population.

## 4. Discussion

The main findings of the current study were as follows: First, the mean %PA after DCA was 44.3%, and the incidence of TLR at 12 months was 3.2% for de novo LM bifurcation lesions after the stentless strategy by DCA followed by DCB angioplasty. Second, IVUS revealed that the lumen and vessel areas increased, while PA and %PA decreased after DCA. Third, the lumen area after DCA was well correlated with the vessel area after DCA; however, %PA after DCA was not correlated with the vessel area after DCA. Fourth, the Δ lumen area and Δ vessel area after DCA were larger in large vessel lesions than in small vessel lesions. However, Δ PA and Δ%PA after DCA were similar between small vessel and large vessel lesions.

A previous study reported that lumen enlargement is the result of a combination of vessel expansion, plaque dissection, and plaque redistribution after balloon angioplasty [11]. On the other hand, plaque removal was the specific mechanism in DCA that is associated with lumen enlargement [6–8], and the effect of plaque removal is controlled by increasing the balloon pressure of the DCA catheter [9]. Generally, large vessel lesions have a large amount of plaque to be debulked; therefore, high balloon pressure of the DCA catheter is required to achieve a lower %PA. Actually, the maximum balloon pressure was greater in large vessel lesions than in small vessel lesions in the current study. Our results revealed that increasing the maximum balloon pressure of the DCA catheter in large vessel lesions was associated with greater vessel expansion but did not increase the effect on plaque reduction compared with that in small vessel lesions. Previous studies have also reported that vessel expansion is a significant contributor to lumen enlargement after DCA [12, 13]. Nakamura et al. demonstrated that the lumen cross-sectional area improved from 2.9 ± 1.5 mm^2^ to 7.0 ± 1.5 mm^2^ (*P* < 0.0001), while the vessel cross-sectional area increased from 17.1 ± 5.9 mm^2^ to 18.7 ± 5.5 mm^2^ (*P* < 0.001) on IVUS after DCA [12]. The largest size (*L*) of the DCA catheter was frequently used (91%) in the current study; therefore, a larger DCA catheter will be necessary for further plaque reduction in large vessel lesions. However, %PA obtained was sufficiently low even in large vessel lesions, and the incidence of TLR at 12 months was acceptable. Accordingly, we consider that the current size (*L*) of the DCA catheter will be adequate for DCA for large vessel LM bifurcation lesions. High balloon pressure of the DCA catheter will strengthen the contribution of vessel expansion in large vessel lesions. However, the DCA catheter is a bulky device and can cause vessel injury. Operators should pay careful attention to the occurrence of dissection, hematoma, and vessel perforation, particularly for large vessel lesions with eccentric plaque or mild calcified plaque when the balloon pressure of the DCA catheter is increased.

### 4.1. Study Limitations

This study has several limitations. First, the sample size was small, and data were analyzed retrospectively. Thus, this should be considered a preliminary study for generating a hypothesis. Second, DCA for the LCX ostium was associated with higher technical challenges during the procedure and higher rates of restenosis than DCA for the LAD ostium. This may have affected the results of the current study. Third, this study was retrospective; therefore, the protocol of the DCA procedure had not been strictly decided. We aimed to obtain %PA < 60% by DCA; however, the number of cuts and maximum balloon pressure were decided by the operator for each case. The DCA procedure itself extremely influenced the vessel response; accordingly, our findings should be validated in other prospective studies. Fourth, we could not evaluate the effect of plaque distal embolization, which was considered as another possible mechanism of lumen enlargement after balloon angioplasty and stent implantation [14, 15]. Minor plaque distal embolization might occur after DCA, but there was no slow flow phenomenon during the procedure and no myocardial infarction after the procedure; lesions with lipid plaque were excluded. We believe that plaque distal embolization might be associated with lumen enlargement after DCA. However, it is quite difficult to evaluate the effect of distal embolization with lumen enlargement using IVUS. Finally, specific techniques are required for interpretation of IVUS findings and for precise control of the DCA catheter during the DCA procedure; therefore; our results may not be generalized.

## 5. Conclusions

The main mechanisms of DCA associated with lumen enlargement are plaque reduction and vessel expansion. High balloon pressure of the DCA catheter was frequently employed to increase the efficacy of plaque reduction particularly in large vessel lesions. However, the effect of plaque reduction did not increase, whereas the contribution to vessel expansion became larger in large vessel lesions after DCA. We should pay careful attention to avoid vessel injury when increasing the maximum balloon pressure of the DCA catheter in large vessel lesions.

## Figures and Tables

**Table 1:** Baseline characteristics of participants.

*Patient characteristics*	31 patients
Age (years)	70 ± 10
Male (%)	29 (94)
Hypertension (%)	24 (77)
Diabetes mellitus (%)	8 (26)
Hyperlipidemia (%)	24 (77)
Hemodialysis (%)	1 (3)
Current smoker (%)	2 (6)
Previous percutaneous coronary intervention (%)	10 (32)
Previous coronary artery bypass graft (%)	0 (0)

*Medication*	
ACE/ARB (%)	21 (68)
*β*-Blocker (%)	22 (71)
Statin (%)	30 (97)
Aspirin (%)	31 (100)
Clopidogrel (%)	10 (32)
Prasugrel (%)	21 (68)

*Medina classification*	
(0, 1, 0) (%)	18 (58)
(0, 0, 1) (%)	4 (13)
(1, 0, 0) (%)	1 (3)
(1, 1, 0) (%)	7 (23)
(1, 1, 1) (%)	1 (3)

*Main target of DCA*	
LAD ostium (%)	21 (68)
LCX ostium (%)	2 (6)
Distal left main trunk (%)	2 (6)
Distal left main trunk and LAD ostium (%)	5 (17)
Distal left main trunk, LAD ostium, and LCX ostium (%)	1 (3)

**Table 2:** Procedural results.

*Directional coronary atherectomy*	32 lesions (31 patients)
Size of the catheter	
ATHEROCUT type M (%)	3 (9)
ATHEROCUT type L (%)	29 (91)
Total number of cuts (times)	28 ± 17
Maximum balloon pressure (atm)	3.5 ± 1.3

*Drug-coated balloon*	
Diameter (mm)	3.3 ± 0.4
Length (mm)	17.5 ± 3.4
Balloon pressure (atm)	8.3 ± 2.9
Procedure time (min)	126 ± 41
Amount of contrast media (mL)	196 ± 72

*Complication during the procedure*	
Vessel perforation (%)	0 (0)
Slow flow phenomenon (%)	0 (0)
Stuck of the DCA catheter (%)	0 (0)

*Procedure-related major events during the hospitalization*	
Death (%)	0 (0)
Emergent TLR or CABG (%)	0 (0)
Myocardial infarction (Q or non-Q) (%)	0 (0)
Access site complications (%)	0 (0)

**Table 3:** Quantitative coronary analysis results.

*Before the procedure*	32 lesions
Minimum lumen diameter (mm)	1.3 ± 0.4
Reference lumen diameter (mm)	3.9 ± 1.1
% diameter stenosis	63.3 ± 10.6
Lesion length (mm)	18.5 ± 6.7

*After the procedure*	
Minimum lumen diameter (mm)	3.4 ± 0.9
Acute gain (mm)	2.0 ± 1.0
Reference lumen diameter (mm)	3.8 ± 1.1
% diameter stenosis	12.2 ± 7.9

*Follow-up*	
Minimum lumen diameter (mm)	3.3 ± 1.1
Late lumen loss (mm)	0.1 ± 0.5
% diameter stenosis	15.4 ± 15.3

**Table 4:** Intravascular ultrasound findings.

*Before directional coronary atherectomy*	32 lesions
Minimum lumen diameter (mm)	1.6 ± 0.3
Lumen area (mm^2^)	3.0 ± 0.9
Vessel area (mm^2^)	13.5 ± 3.6
Plaque area (mm^2^)	10.5 ± 3.3
% plaque area	77.5 ± 6.1

*After directional coronary atherectomy*	
Minimum lumen diameter (mm)	2.8 ± 0.3
Lumen area (mm^2^)	8.9 ± 2.1
Vessel area (mm^2^)	16.1 ± 3.8
Plaque area (mm^2^)	7.2 ± 2.3
% plaque area	44.3 ± 6.7
Intimal dissection (%)	5 (15.6)
Medial dissection (%)	0 (0)
Hematoma (%)	0 (0)

## Data Availability

The data used to support the findings of this study are restricted by the Ethics Committee of Saiseikai Yokohama-City Eastern Hospital in order to protect patient privacy and are available from the corresponding author upon request.
